# Supplementary material for: Non-adaptive measurement-based quantum computation on IBM Q
Source: Sci Rep. 2023 Sep 18;13:15428. doi: 10.1038/s41598-023-41025-4 (PMC10507095; doi:10.1038/s41598-023-41025-4)
Supplement: Supplementary file 1 — Supplementary Information. [file 41598_2023_41025_MOESM1_ESM.pdf]

# Supplemental Information
# Non-adaptive measurement-based quantum computation on IBM Q

**Jelena Mackeprang**[1,2,+], **Daniel Bhatti**[1,2], **and Stefanie Barz**[1,2,*]

[1]Institute for Functional Matter and Quantum Technologies, University of Stuttgart, 70569 Stuttgart, Germany
[2]Center for Integrated Quantum Science and Technology (IQST), University of Stuttgart, 70569 Stuttgart, Germany
[+]Present address: QuSoft and Centrum Wiskunde & Informatica (CWI), Science Park 123, 1098 XG Amsterdam, The Netherlands
[*]stefanie.barz@fmq.uni-stuttgart.de

## Additional proofs

### Proof of efficient computability of $h_k(x)$ for all $k$

Here, we prove the following:

**Theorem 1.** *To deterministically compute the k-bit function $h_k(x)$, as defined in Eqn. (11) in the main text, one only requires $l = k+1$ qubits forming the l-qubit generalised GHZ state [see Eqn. (1) in the main text], the pre-processing defined in Eqn. (12) in the main text and the measurement settings $\hat{m}_i(s_i = 0) = X$, $\hat{m}_i(s_i = 1) = Y$ $\forall i$.*

*Proof.* This can easily be proven via natural induction. We start by observing the following:

$$h_{k+1}(x) = \bigoplus_{i=0}^{k-1} \bigoplus_{j=i+1}^{k} x_i x_j \oplus \bigoplus_{i=0}^{k} x_i \tag{S1}$$

$$= \bigoplus_{i=0}^{k-2} \bigoplus_{j=i+1}^{k-1} x_i x_j \oplus x_k \left( \bigoplus_{i=0}^{k-1} x_i \right) \oplus \bigoplus_{i=0}^{k-1} x_i \oplus x_k \tag{S2}$$

$$= h_k(x) \oplus x_k \left( \bigoplus_{i=0}^{k-1} x_i \right) \oplus x_k, \tag{S3}$$

meaning that we can write $h_{k+1}(x)$ as a sum of $h_k(x)$ and a term that is only dependent of $x_k$ and the sum over the first $k$ bits of $x$, i.e. $x_0, x_1, \ldots, x_{k-1}$. We abbreviate this sum by $S$:

$$S = \bigoplus_{i=0}^{k-1} x_i. \tag{S4}$$

We will now start with the actual proof by induction. For this, we need the general condition for NMQC to be successful. In Ref.[1], it is shown that any Boolean function can be computed with NMQC when using the generalised GHZ state and the general measurement operators:

$$\hat{m}_i(s_i) = \cos(\phi_i s_i) X + \sin(\phi_i s_i) Y. \tag{S5}$$

The condition that must be fulfilled for the deterministic computation of a function $f : \{0,1\}^n \to \{0,1\}$ is[1]:

$$e^{i \sum_{j=0}^{l-1} s_j \phi_j} = (-1)^{f(x)+c}, \tag{S6}$$

where $\phi_j \in (-\pi, \pi)$ are angles yet to be determined, $c$ is a bit that can be added in post-processing and $s_j$ is related to the input bit string $x$ by the pre-processing $s = (Px)_\oplus$. To prove theorem 1 by natural induction, we first show that condition (S6) holds for $h_3(x)$ and then show that it is fulfilled when $k \mapsto k+1$. As $h_k(x)$ is zero for $x = 0$ for all $k$, we can set $c$ in Eqn. (S6) to zero.

Additionally, we know that $\phi_j = \pi/2\ \forall j$ due to the fixed measurement settings. This, combined with the pre-processing given by Eqn. (12) of the main text simplifies Eqn. (S6) for $h_k(x)$ to:

$$e^{i\frac{\pi}{2}\left(\sum_{j=0}^{k-1} x_j + S\right)} = (-1)^{h_k(x)}, \tag{S7}$$

where $S$ is the abbreviation for the sum modulo 2 of all $k$ bits in $x$ [see Eqn. (S4)].

1. $k = 3$. We list the values for $\sum_{j=0}^{k-1} x_j$, $S$, $h_3(x)$ and $e^{i\frac{\pi}{2}\left(\sum_{j=0}^{k-1} x_j + S\right)}$ for all 8 input strings in Table S1, of which one can read of that the induction hypothesis is fulfilled.
2. $k \mapsto k+1$. When increasing $k$ by 1, the r.h.s of Eqn. (S7) becomes:

$$(-1)^{h_{k+1}(x)} = (-1)^{(h_k(x) \oplus x_k S \oplus x_k)}, \tag{S8}$$

where we have inserted Eqn. (S3). According to condition (S7), for deterministic NMQC to function for $k \mapsto k+1$, the following must hold:

$$(-1)^{(h_k(x) \oplus x_k S \oplus x_k)} = (-1)^{h_k(x)} \cdot (-1)^{x_k S \oplus x_k} \tag{S9}$$

$$= e^{i\frac{\pi}{2}\left(\sum_{j=0}^{k-1} x_j + S\right)} \cdot (-1)^{x_k S \oplus x_k} \tag{S10}$$

$$= e^{i\frac{\pi}{2}\left(\sum_{j=0}^{k} x_j + (S \oplus x_k)\right)}. \tag{S11}$$

We have inserted the induction hypothesis (S7) in Eqn. (S10). In summary, we must show that this:

$$e^{i\frac{\pi}{2}\left(\sum_{j=0}^{k-1} x_j + S\right)} \cdot (-1)^{x_k S \oplus x_k} = e^{i\frac{\pi}{2}\left(\sum_{j=0}^{k} x_j + (S \oplus x_k)\right)} \tag{S12}$$

is true for all $x$. We can cancel out $e^{i\frac{\pi}{2}\left(\sum_{j=0}^{k-1} x_j\right)}$ on both sides and are left with:

$$e^{i\frac{\pi}{2} S} \cdot (-1)^{x_k S \oplus x_k} = e^{i\frac{\pi}{2}(x_k + (S \oplus x_k))}. \tag{S13}$$

Eqn. (S13) only depends on $x_k$ and $S$. The final steps of this proof thus merely consist of checking if it is correct for the four possible combinations of $x_k$ and $S$. For both $x_k = 0$ and $S = 0$, both sides of the equation are equal to 1. For $x_k = 1$ and $S = 0$, the l.h.s. becomes $(-1)$ and the r.h.s becomes $e^{i\frac{\pi}{2}(1+1)} = e^{i\pi}$. For $x_k = 0$ and $S = 1$, the l.h.s. equals $e^{i\frac{\pi}{2}}$ and the r.h.s. turns into $e^{i\frac{\pi}{2}(0+1)}$. Lastly, for $x_k = S = 1$, the l.h.s. is $i$ and the r.h.s. is equal to $e^{i\frac{\pi}{2}(1+0)} = i$.

This completes the proof of theorem 1.

□

Note that, using the appropriate measurement settings and pre-processing, the NMQC output will always be $h_k(x)$, which means that any probabilistic NMQC game using the same measurement settings and pre-processing (and the GHZ state as a computational resource) has a quantum success probability of 1, translating to a bound $q = 1$ of the associated Bell inequality.

| $x$ | (0,0,0) | (0,0,1) | (0,1,0) | (0,1,1) | (1,0,0) | (1,0,1) | (1,1,0) | (1,1,1) |
|---|---|---|---|---|---|---|---|---|
| $\sum_{j=0}^{k-1} x_j$ | 0 | 1 | 1 | 2 | 1 | 2 | 2 | 3 |
| $S$ | 0 | 1 | 1 | 0 | 1 | 0 | 0 | 1 |
| $h_3(x)$ | 0 | 1 | 1 | 1 | 1 | 1 | 1 | 0 |
| $e^{i\frac{\pi}{2}\left(\sum_{j=0}^{k-1} x_j + S\right)}$ | $e^0 = 1$ | $e^{i\frac{\pi}{2}\cdot 2} = -1$ | $-1$ | $e^{i\frac{\pi}{2}(2+0)} = -1$ | $-1$ | $-1$ | $-1$ | $e^{i\frac{\pi}{2}(3+1)} = 1$ |

**Table S1.** Table to test induction hypothesis for $h_3(x)$

### Classical bound of the probabilistic NMQC game $h_k(x)$ for all $k$

Here, we explain how to obtain the classical success probabilities of the probabilistic NMQC games induced by $h_k(x)$ with a uniform sampling distribution $\xi(x) = 1/2^k$. To be precise, we prove the following theorem:

**Theorem 2.** *The LHV bound c of the Bell inequality bounding the average success probability of the probabilistic NMQC game [according to Eqn. (2) of the main text] induced by the function $h_k(x)$ defined in Eqn. (11) in the main text with a uniform sampling distribution $\xi(x) = 1/2^k$ is equal to $c = 2^{-\frac{k}{2}}$ for even k and $c = 2^{-\left(\frac{k-1}{2}\right)}$ for odd k.*

*Proof.* The proof entirely consists of combining one simple observation with previous knowledge on the non-linearity of Boolean functions. As, by convexity, the best LHV strategy in an NMQC game with a uniform sampling distribution $\xi(x) = 1/2^n$, where the goal is to compute a Boolean function $f : \{0,1\}^n \to \{0,1\}$, is to output the *closest* linear function. The average classical success probability $\bar{p}_S^c$ is given by:

$$\bar{p}_S^c = \left[2^n - \min_{g\,\text{linear}} \text{dist}(f,g)\right]/2^n, \tag{S14}$$

where the distance $\text{dist}(f,g)$ of two Boolean functions $f$ and $g$ is their Hamming distance, i.e. the number of arguments $x$ for which $f(x) \neq g(x)$.

As already pointed out in Ref.[2], the minimum distance of a function $f$ to the closest linear function $g$ is its *nonlinearity* NL. Boolean functions with the maximum nonlinearity possible are called *bent* functions[3,4]. They lead to a minimal classical success probability $\bar{p}_S^c$ and are thus best suited to demonstrate non-locality with this type of NMQC game (see also Ref.[2]).

The function $h_k(x)$, as defined by Eqn. (11) in the main text is in fact one of the few symmetric bent functions[3,4]. For even $n$, its non-linearity is $\text{NL} = 2^{n-1} - 2^{n/2-1}$ (see Ref.[3]). For odd $n$, its non-linearity is $\text{NL} = 2^{n-1} - 2^{(n-1)/2}$. Inserting these values into Eqn. (S14) and using Eqn. (2) of the main text, one then immediately obtains the classical bounds of the associated Bell inequalities.

□

Note that, as the quantum bound of the Bell inequality $q$ is always 1, this NMQC game is related to a Bell inequality, for which the ratio $q/c$ between the quantum bound and its LHV counterpart increases exponentially.

## Individual measured bounds

To understand why the averaged results are notably smaller than the maximal ones produced by a single configuration we will take a look at all individual measured bounds, i.e. all specific qubit configurations, of two Bell inequalities. Fig. S1 shows the (mitigated) bounds of the Bell inequality induced by $\text{OR}_3^{\oplus}(x)$ (see Table 1 in the main text) for every single four-qubit configuration. One can see that the measured bounds strongly vary, even including negative values. This coincides with the measured expectation values for a qubit configuration, which produced high values (0-1-2-3), and a qubit configuration, which produced negative values (10-18-12-15) (see Fig. S3).

In general, the performance of single qubits varies over time. The experiment for $\text{OR}_3^{\oplus}(x)$ was run on the 7th June 2022, whereas the one for $h_3(x)$ was run on 21st May 2022. At the time of the four outlier NMQC runs for $\text{OR}_3^{\oplus}(x)$, the readout-error rates of the qubits 18 and 12 were 0.011 and 0.022 compared to 0.017 and 0.008 for the same qubits during the measurements belonging to the probabilistic NMQC game induced by $h_3(x)$. We plot the violations of the associated Bell inequality for $h_3(x)$ in Fig. S2, where no qubit configuration exhibits this kind of behaviour. Therefore, to show the violation of Bell inequality for a single qubit configuration one has to perform multiple runs at different times and average the results.

## References


1. Hoban, M. J., Campbell, E. T., Loukopoulos, K. & Browne, D. E. Non-adaptive measurement-based quantum computation and multi-party Bell inequalities. *New J. Phys.* **13**, 023014, DOI: 10.1088/1367-2630/13/2/023014 (2011).

2. Raußendorf, R. Contextuality in measurement-based quantum computation. *Phys. Rev. A* **88**, 022322, DOI: 10.1103/PhysRevA.88.022322 (2013).

3. Savický, P. On the bent Boolean functions that are symmetric. *Eur. J. Comb.* **15**, 407–410, DOI: https://doi.org/10.1006/eujc.1994.1044 (1994).

4. Maitra, S. & Sarkar, P. Maximum nonlinearity of symmetric Boolean functions on odd number of variables. *IEEE Transactions on Inf. Theory* **48**, 2626–2630, DOI: 10.1109/TIT.2002.801482 (2002).

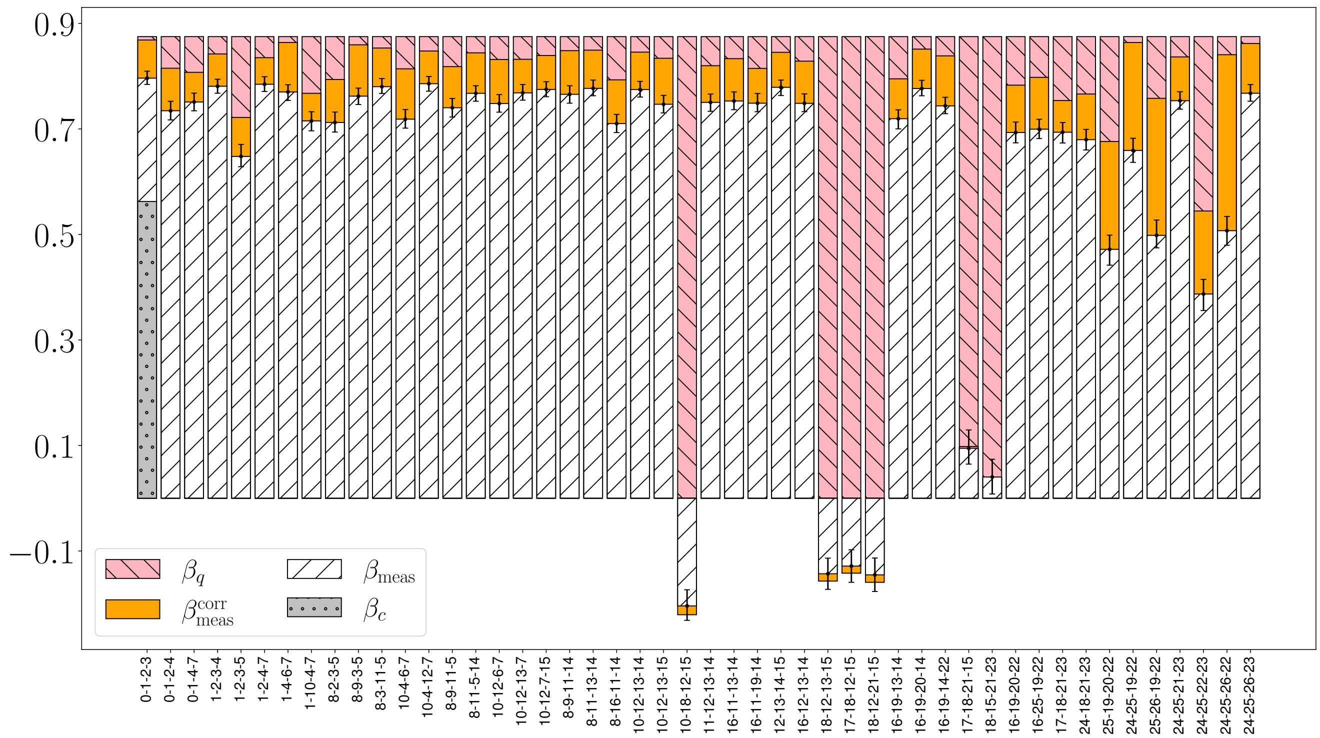


**Figure S1.** (Mitigated) measured bounds of the Bell inequality for every qubit configuration induced by the functions $\text{OR}_3^{\oplus}(x)$.

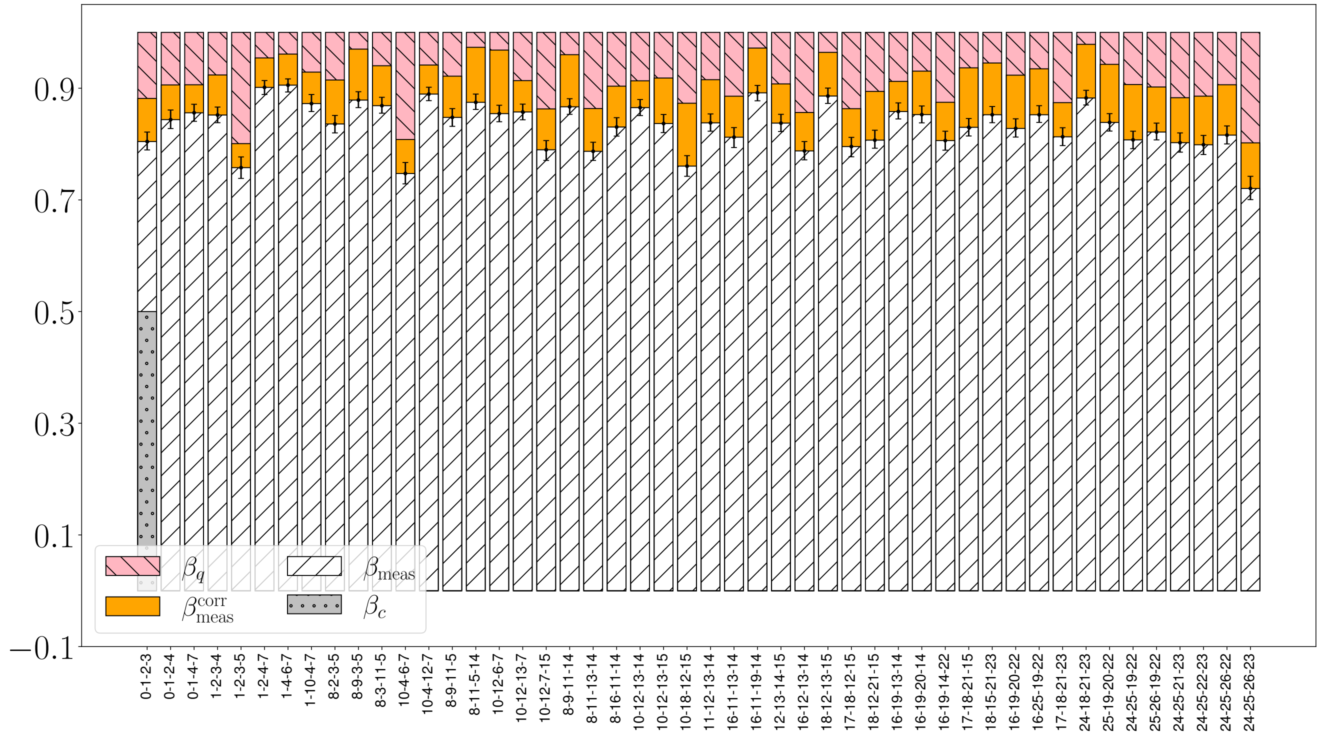


**Figure S2.** (Mitigated) measured bounds of the Bell inequality for every qubit configuration induced by the functions $h_3(x)$.

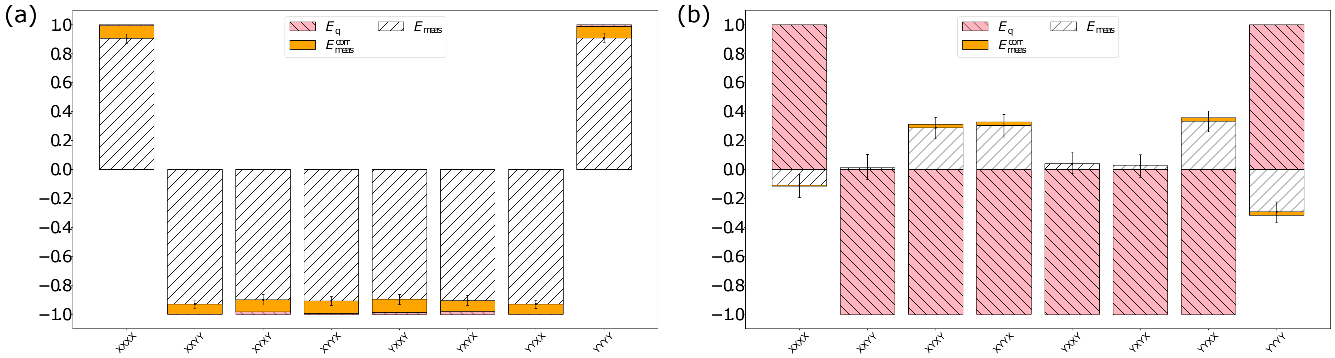


**Figure S3.** Individual expectation values of the operators making up the Bell operator induced by the probabilistic NMQC game for the function $OR_3^{\oplus}$ for two different qubit configurations. In theory, they should all be $\pm 1$. (a) Qubit configuration 0-1-2-3. (b) Qubit configuration 10-18-12-15.
